# Remission of Thymoma on Steroid Therapy in a Patient With Atypical Thymoma-Associated Multiorgan Autoimmunity: A Case Report and Literature Review

**DOI:** 10.3389/fimmu.2021.584703

**Published:** 2021-04-29

**Authors:** Ewa Wrona, Sylwia Dębska-Szmich, Marta Pastuszka, Marcin Braun, Rafał Czyżykowski, Piotr Potemski

**Affiliations:** ^1^ Department of Chemotherapy, Copernicus Memorial Hospital in Lodz, Medical University of Lodz, Lodz, Poland; ^2^ Department of Dermatology, Medical University of Lodz, Lodz, Poland; ^3^ Department of Pathology, Chair of Oncology, Medical University of Lodz, Lodz, Poland

**Keywords:** thymoma, thymoma-associated multiorgan autoimmunity (TAMA), paraneoplastic autoimmune multiorgan syndrome (PAMS), mucocutaneous lesions, remission on steroids

## Abstract

In up to 34% of cases, thymoma, itself a rare neoplasm, is accompanied by autoimmune disorders, two of which are thymoma-associated multiorgan autoimmunity (TAMA) and paraneoplastic autoimmune multiorgan syndrome (PAMS). Unfortunately, differential diagnosis between these two entities can be challenging since no strict PAMS definition exists and PAMS can overlap with a subgroup of TAMA patients with skin lesions as leading presentation. We present a case of a 68-year-old woman with a diagnosis of thymoma accompanied by myasthenia gravis, hypothyroidism and GvHD-like mucocutaneous lesions that initially could account to both TAMA and PAMS diagnosis. However, following the exclusion of humoral autoimmunity against components of epithelial cells junction, TAMA was finally established. Interestingly, the introduction of corticosteroid therapy for TAMA symptom management resulted in unexpected partial remission of thymoma with no impact on mucocutaneous lesions. Our case study is an example of two extremely rare phenomena accompanying thymomas: unprecedented TAMA presentation with GvHD-like mucositis, which as we postulate should be placed in the spectrum of TAMA, and tumor remission on steroids.

## Introduction

Thymoma is described as a rare neoplasm that constitutes roughly 0.2% to 1.5% of all malignancies, with an annual age-standardized incidence rate of 0.13 cases per 100 000 population (1). Up to 34% of thymomas are thought to be accompanied by symptoms of autoimmunity (2). Based on previous reports, the most frequently described ones are myasthenia gravis (MG) (96.5%), pure red blood cell aplasia (PRCA) (2.2%) and hypogammaglobulinemia (0.6%); while panencephalitis, enterocolitis, autoimmune hepatitis and erythematous dermatitis are incidentally described in the literature (2–7). The autoimmune reactions that accompany thymoma have a number of possible targets, resulting in a wide range of symptoms; in addition, as the patients demonstrate high heterogeneity there is great disagreement on how to classify these rare cases.

The term ‘thymoma-associated multiorgan autoimmunity’ (TAMA) emerged in 2007 to describe patients with Graft-versus-Host Disease–like (GvHD-like) dermatitis, enterocolitis or hepatitis with underlying thymoma and no prior haematopoietic stem cell (HSCT) nor organ transplantation (8). This contrasted with the term ‘paraneoplastic autoimmune multiorgan syndrome’ (PAMS), which was proposed in 2001 for cases characterized by mucocutaneous lesions often extending to the aerodigestive tract with bronchial involvement, humoral autoimmunity against cellular adhesion molecules, poor response to treatment and high mortality rate (9, 10). TAMA criteria may apply more closely to a narrow, well-distinguished group of thymoma patients, currently represented by only 31 published cases; however, PAMS is much broader and has a distorted definition and its diagnostic PAMS criteria can partially overlap with patients suspected of TAMA with predominant dermatitis (**Supplementary Table 1**). Moreover, several groups postulate TAMA, as well as paraneoplastic pemphigus to be a variation of PAMS, however this opinion is not widely approved (11, 12).

We report a case of a patient diagnosed with locally-advanced thymoma accompanied by MG, hypothyroidism, slowly progressing mucocutaneous lesions of mild intensity with histopathology features classified as GvHD-like, and neither humoral autoimmunity nor bronchial involvement. The article describes the extensive diagnostic process employed to resolve a lack of consensus on how to properly distinguish TAMA from PAMS, two extremely rare entities. Strikingly, the use of corticosteroid therapy, as a treatment of persistent mucocutaneous lesions, resulted in partial remission of thymoma with no improvement of skin or oral cavity condition. Which altogether led us to transfer the discussion on our patient to a wider audience.

## Case Description

**Figure 1:**
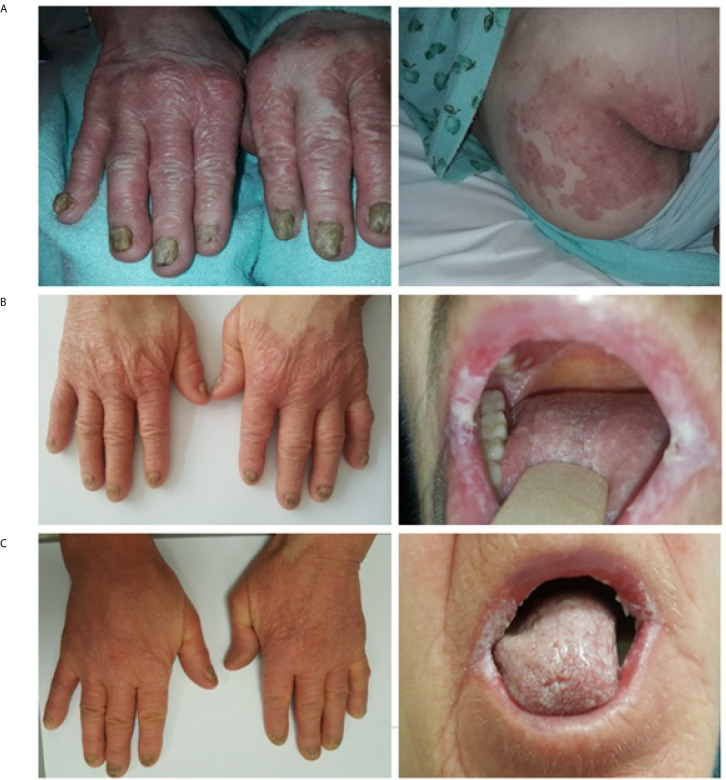
TAMA. Skin and mucosal lesions photographed **(A)** at the onset of chemotherapy, **(B)** after six cycles of systemic treatment and **(C)** after three months of prednisone therapy.

**Figure 2:**
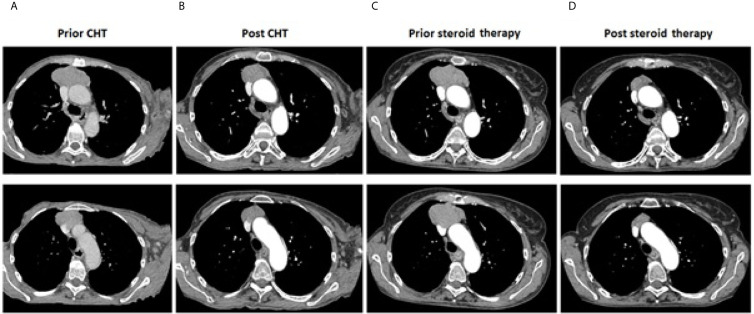
Thymoma. Computer tomography imaging showing maximal tumor dimensions (upper row) and infiltration on VCS (bottom row): **(A)** prior systemic treatment (64 x 30 x 76 mm); **(B)** after completion of chemotherapy (51 x 26 x 70 mm); **(C)** prior corticosteroids implementation (65x63x75mm); **(D)** after three months of corticosteroid therapy (30x17mm).

A 68-year-old Caucasian woman with an initial diagnosis of thymoma was admitted to the Department of Chemotherapy in November 2018 for an evaluation and treatment implementation. On admission, she reported a six-month history of poor appetite, weight loss of 12 kg (24% of initial body weight), generalized fatigue, muscle weakness, oral cavity lesions resembling candidiasis that persist despite antifungal therapy, erythematous scaly papules affecting skin surrounding her nose, mouth and buttocks, as well as the distal parts of the upper extremities and palms, with concomitant degeneration of the finger nail plates (**Figure 1A**). No chest pain, cough, dyspnea or diarrhea was reported, and no signs of phrenic nerve palsy were observed. No relevant family history was reported. The patient suffered from hypertension, otherwise healthy. Her performance status (PS) according to Eastern Cooperative Oncology Group (ECOG) was assessed as 4, mostly due to severe muscle weakness. Computed tomography (CT) revealed a locally advanced tumor (64 x 30 x 76 mm) in the upper part of the anterior mediastinum with suspected infiltration of superior vena cava (VCS); no signs of distant metastases nor lymph node involvement were observed (**Figure 2A**). Histopathology examination revealed WHO type B1 thymoma with immunophenotype: AE1/AE3+, BCL6 -/+, CD20-, CD23-, CD3+, CD5+, cyclin D1-, p53 -, Ki67 high. The disease was assessed as stage III according to Masaoka-Konga and as rT3N0M0 according to IASCL/ITMIG.

## Diagnostic Assessment

### Autoimmunity Management

The patient’s complete blood count, liver and kidney function profiles on admission were within normal limits. Neither hypogammaglobulinemia nor pure red blood cell aplasia were suspected. The patient’s thyroid profile normalized gradually on levothyroxine sodium (75 μg once daily) supplementation and Hashimoto thyroiditis, as a cause of primary hypothyroidism, was ruled out (GH, PRL levels were within normal limits, anti-TPO and anti-TG antibodies were negative). Additionally, MG diagnosis was confirmed based on the presence of anti-AchR antibodies (196 nmol/l in RIA assay) with a lack of anti-MuSK antibodies (<0.03 nmol/l in RIA assay). Muscle weakness gradually resolved upon pyridostigmine bromide treatment (60 mg three times a day) and physical therapy.

### Thymoma Treatment

Taking into consideration the patient’s poor performance status (resulting mainly from uncontrolled MG and hypothyroidism symptoms) and cachexia, the treatment plan involved reduced dose chemotherapy, symptom control and physical therapy while thymectomy and radical radiotherapy were contraindicated due to VCS infiltration. First-line PE combination chemotherapy (cisplatin 60 mg/m2, etoposide 75 mg/m2 day 1 to 3 for 6 cycles every 3 weeks) was launched with 20% dose reduction only during the first cycle. Chemotherapy was well tolerated, with no severe adverse events (≥Grade 3 according to Common Terminology Criteria for Adverse Events (CTCAE) version 5.0).

During the systemic treatment, symptoms mitigated and patient's condition gradually improved, however with no signs of reduction of mucocutaneous lesions (**Figure 1B**). A CT scan after six cycles of chemotherapy showed that the longest diameter of the tumor slightly decreased from 76 mm to 70 mm (**Figure 2B**). Despite demonstrating significant clinical improvement (PS1 – marginal muscle weakness, persistent mucocutaneous lesions) and achieving stable disease (SD) according to Response Evaluation Criteria in Solid Tumors (RECIST) 1.1 criteria, neither thymectomy nor radical radiotherapy were advised due to persistent VCS involvement.

### Skin and Oral Mucosa Lesions

**Figure 3:**
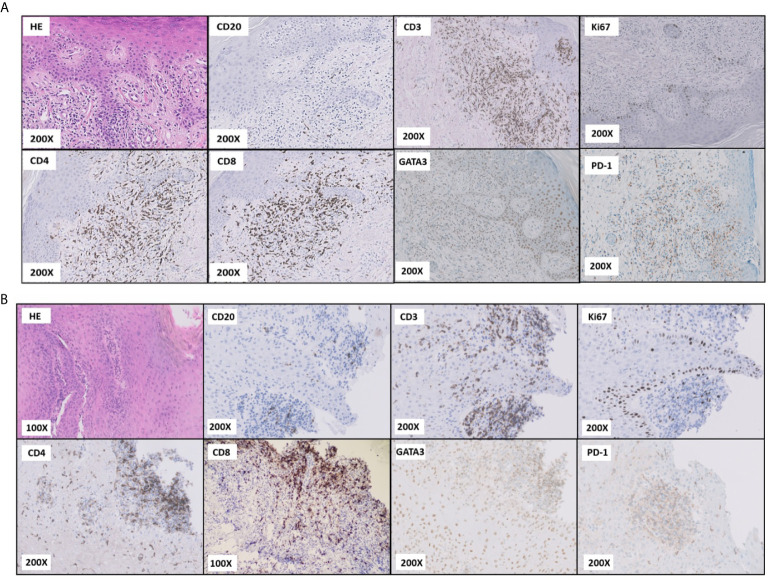
TAMA. HE and immunostaining of the skin **(A)** and oral mucosa **(B)** samples in magnification.

To diagnose the mucocutaneous lesions, surgical samples of affected tissues were obtained. Based on histopathological features, immunohistochemistry and clinical data, various GvHD-like changes were observed in the skin: parakeratosis, dyskeratosis, intensive basal layer destruction, intracellular oedema, subepidermal atypic lymphocyte T infiltration without features of malignancy (CD3+, CD4+>CD8+ at a ratio of 1.5-2:0, incidental CD30+), and focal destruction of skin appendages (**Figure 3A**) despite the lack of HSCT or organ transplantation in the patient’s past medical history. Correspondingly, GvHD-like changes were also reported in the oral mucosa sample: intensive T-cell infiltration (CD3+, CD4+>CD8+ at a lower ratio than in skin sample, incidental B-cell lymphocytes), basal layer destruction, acanthosis, hyperkeratosis (**Figure 3B**). Blood serum evaluation was negative for RF, anti-CCP, anti-Ro, anti-La and anti-dsDNA and weakly positive for anti-Sm antibodies.

Based on the test results and clinical presentation, a strong suspicion of TAMA was raised. However, due to the fact that diagnostic criteria for TAMA and PAMS overlap for patients with the GvHD-like dermatitis, and that mucosal involvement was found to be more characteristic of PAMS, the differential diagnosis was broadened. An analysis of humoral autoimmunity against epithelial cells junction components was performed (**Supplementary Table 1**). Ultimately, the skin and blood samples tested negative in direct immunofluorescence (DIF) for IgG, IgM, IgA and C3 deposits, and negative in indirect immunofluorescence (IIF) on a rat urinary bladder. In addition, the patient was negative for antibodies against desmoglein 1, desmoglein 3, BP 180, BP 230, envoplakin and collagen type VII (all equal or less than 0.1 RU/ml, with cut-off >1.0 RU/ml in ELISA assay); this definitively ruled out a diagnosis of PAMS. Therefore, seven months after chemotherapy completion, low dose prednisone (0.5 mg/kg a day) treatment was launched.

Unfortunately, neither skin nor mucosal lesions resolved after three months of low-dose prednisone therapy (**Figure 1C**); no other anti-cancer treatment was administered and dosing of 0.5 mg/kg a day was not escalated due to poor tolerance. Quite unexpectedly, routine follow-up CT scan revealed good partial response (PR) of the upper-mediastinal mass, according to RECIST 1.1 criteria, with its measurements decreasing substantially to 30 mm from 75 mm prior to corticosteroid therapy, and the disappearance of VCS infiltration (**Figures 2C, D** **)**.

Due to resolved VCS infiltration observed in the CT scan, radical thymectomy was advised; however, the patient refused the surgery. She has since been referred to the Department of Radiotherapy for irradiation of residual thymoma with 66 Gy (33 x 2 Gy). At the time of writing, the patient remained in PR (tumor measurements on the latest CT scan 30 x 24 x 38 mm) with persistent mucocutaneous lesions, negligible muscle weakness, stable body weight and energy level. Due to poor tolerance (dizziness, headache, nausea), the dose of prednisone was decreased to 0.3 mg/kg a day with no deterioration of skin condition and no signs of thymoma progression. Levothyroxine, pyridostigmine and concomitant physical therapy were maintained. Detailed timeline on the patient’s diagnostic process and treatment was presented in the **Supplementary Figure 1**.

## Discussion

Including the patient described in this article, only 32 cases of TAMA were found to have been published between 1995 and 2020 (8, 13–36). Twenty six patients presented with GvHD-like dermatitis, clinically manifested as erythematous scaly papules disseminated over the whole body (**Table 1**). Corticosteroid therapy, both oral and topical, caused skin lesion remission in twelve cases out of 19; while phototherapy was much more effective, but rarely applied (four remissions out of five described cases). Oral mucosa lesions, clinically demonstrated as erosions and ulcerations, were described only in four TAMA patients; however, no biopsy sample was obtained to determine their histological presentation.

**Table 1:** A summary of all known published cases described as Thymoma-associated multiorgan autoimmunity.

Pt no.	Age at diagn.	Gender	Thymoma type/stage	Clinical symptoms	Other thymoma associated autoimmunities	Diagnosis	TAMA treatment	Prognosis	Reference
1	50	Male	IV	Erythematous scaly rash of the face, trunk, upper and lower extremities, palms, feet, no scalp or mucosal involvement edema, diarrhea	Hypogammaglobulinemia	GvHD-like changes in the skin biopsy sample	Steroids, topical triamcinolone, PUVA, IVIG	PUVA was applied only few times, too little to draw any conclusions. Died 3 years after first TAMA symptoms due to sepsis.	Wadhera et al. (8)
2	20	Male	B1, recurrent	Severe diarrhea, weight loss	–	GvHD-like features in colon biopsy sample	Steroids	Several remissions of thymoma entailed by TAMA symptoms resolving	Kornacki et al. (17)
3	38	Female	IVa	Erythematous morbilliform eruption of the skin on trunk, upper and lower extremities, oral mucosa ulcerations, abnormal LFTs, diarrhea	MG, hypogammaglobulinemia	GvHD-like changes in the skin and colon biopsy samples	Steroids	Died of diarrhea and respiratory infection	Wang et al. (13)
4	47	Male	A I	Pruritic morbilliform eruption on face and trunk, mucosal erosions, diarrhea	Hypogammaglobulinemia, PRCA	GvHD-like changes in the skin biopsy sample	IVIG, cyclosporine A	No data	Holder et al. (19)
5	35	Female	IVa	Morbilliform eruption on trunk, diarrhea	MG	GvHD-like changes in the skin, duodenum and colon biopsy sample	Steroids, cyclosporine A, IVIG, octreotide (for diarrhea)	Partial improvement of skin lesions on steroids. Diarrhea moderately improved on octreotide. Died of thymoma progression	Lowry et al. (16)
6	46	Male	IVa	Diarrhea	–	GvHD-like changes in the colon biopsy sample	Steroids	Persistent diarrhea	Sader et al. (31)
7	57	Female	III	Diarrhea, erythema-multiforme resembling skin lesions, abnormal LFTs	Hypogammaglobulinemia	GvHD-like changes in the skin biopsy sample	Steroids, IVIG	Died of sepsis following complications of thymectomy.	Sleijfer et al. (24)
8	26	Male	IVa	Erythematous papules on the trunk, face, upper and lower extremities, palms, soles, diarrhea	MG	GvHD-like changes in the skin biopsy sample	Steroids, cyclosporine A, plasmapheresis, IVIG, mechanical ventilation (due to MG crisis)	Mild improvement of skin lesions after steroids and cyclosporine. Died in respiratory failure	Gupta et al. (21)
9	52	Male	IVb	Diarrhea	MG	GvHD-like phenotype in the biopsy samples from the stomach, small intestine and colon. DIF positive	–	Diarrhea resolved after thymoma resection. Died of respiratory insufficiency due to MG crisis	Mais et al. (22)
10	48	Female	IV	Refractory diarrhea, cachexia	MG	GvHD-like phenotype in the specimen from the small bowel	Steroids, IVIG, plasmapheresis, intubation (for MG crisis)	No improvement of diarrhea. Died of respiratory distress due to MG crisis and K.pneumonia positive pneumonitis and following DIC	
11	40	Female	B1, recurrent as B2	Erythroderma of the trunk, lower and upper extremities	MG	GvHD-like changes in the skin biopsy sample	Steroids, tacrolimus, cyclosporine A	Mild improvement of skin lesions after 2 mo. on steroids and cyclosporine A. While decreasing steroids doses erythroderma exacerbation and MG crisis. Died in respiratory failure and sepsis	Nakagiri et al. (33)
12	20	Male	B1, recurrent	Diarrhea	–	GvHD-like changes in colonic biopsy specimen along first time diagnosis of thymoma and concomitantly with its recurrence 2 years after.	Steroids, cyclosporine A	Diarrhea resolved multiple times concomitantly with thymoma remission	Offerhaus et al. (32)
13	39	Female	AB, recurrent	Skin lesions, abnormal LFTs, diarrhea	Sjögren's syndrome, PRCA, autoimmune thrombocytopenia	GvHD-like colitis and duodenitis, GvHD-like changes in skin and liver biopsy samples	Cyclosporine A, anti-CD3 antibodies	Died of respiratory insufficiency	
14	61	Male	AB, recurrent	Diarrhea	–	GvHD-like enterocolitis	No data	No data	
15	Early 50s	Female	recurrent	Widespread pruritic, erythematous lesions on the face, trunk, upper and lower extremities	–	GvHD-like changes in the skin biopsy sample	Steroids, PUVA, mycophenolate mofetil	Skin lesions improving on steroids and further after adding PUVA	Gishen et al. (28)
16	50	Male	AB, recurrent	Erythroderma of the face, trunk, upper and lower extremities, abnormal LFTs	MG	GvHD-like changes in the skin biopsy sample	Steroids, tacrolimus	Several MG crisis requiring mechanical ventilation, thymoma regression on CHT entailed improvement of skin lesions.	Nagano et al. (34)
17	64	Female	disseminated	Pruritic erythema distributed over the face, trunk, upper and lower extremities	–	DIF negative GvHD-like changes in the skin biopsy sample	Steroids, cyclosporine A, NB-UVB	Steroids and cyclosporine A with no impact on skin lesions. Good response to steroids +NB-UVB. Died of aspergillosis 5 month after TAMA diagnosis	Murata et al. (35)
18	40	Female	B1, recurrent	Psoriasiform erythroderma, diarrhea, abnormal LFTs	MG	GvHD-like changes in the skin biopsy sample	Steroids, cyclosporine A, tacrolimus	Skin lesions improved after 2 mo. of steroid therapy, but reappeared after steroids discontinuation. Died of sepsis 5 mo. after TAMA diagnosis	Hanafusa et al. (20)
19	36	Female	B1	Erythroderma	MG	GvHD-like changes in the skin biopsy sample	Steroids, tacrolimus, ambenonium	Improved skin condition on oral and topical steroids, recurred after treatment withdrawal. Died 3y after TAMA diagnosis of pneumonia	
20	42	Female	B2, disseminated	Generalized psoriasiform erythroderma and oral mucosa erosions, abnormal LFTs	MG	GvHD-like changes in the skin biopsy sample	Steroids	Skin lesions resolved on steroids, recurred after steroid withdrawal. Treatment was discontinued after massive progression of thymoma	
21	69	Female	B2, III	Erythema involving the face, trunk, upper and lower extremities	–	GvHD-like changes in the skin biopsy sample	Topical steroids	Skin lesions resolved after radical thymoma resection	Motoishi et al. (14)
22	50	Female	B1, recurrent	Erythematous pruritic lesions on the trunk, upper and lower extremities, abnormal LFTs	MG with multiple crisis	GvHD-like changes in the skin biopsy sample	Steroids	Died of respiratory insufficiency due to MG crisis	Warren et al. (25)
23	32	Male	B2, recurrent	Scaly annular erythema of the trunk developing into erythroderma	MG, cardiomyopath, opportunistic infections	GvHD-like changes in the skin biopsy sample	Steroids, NB-UVB	No improvement of skin condition on steroids. Skin lesions responsive to NB-UVB. Died of sepsis.	Nakayama et al. (27)
24	58	Male	B3, IVa	Pruritic eruptions	MG, PRCA	GvHD-like changes in the skin biopsy sample	Steroids, cyclosporine A, phototherapy	Partial improvement on steroids and phototherapy. Died suddenly due to cardiac arrest	Shiba et al. (30)
25	72	Male	B1	Erythemous eruptions	PRCA	GvHD-like changes in the skin biopsy sample	Steroids	Skin lesions dissolved on steroids. Died of “disturbance of consciousness”	
26	61	Male	disseminated	Recurrent oral mucosal and lip erosions, erythematous, scaling nonpruriting plaques of the trunk, upper and lower extremities		DIF negative GvHD-like changes in the skin biopsy sample	Steroids, azathioprine	No improvement of oral mucositis on steroids and azathioprine, skin lesions improved on steroids.	Hung et al. (18)
27	52	Female	Locally advanced	Scaly erythema, red papules across the trunk, upper and lower extremities evolving into erythema, oral mucosa erosions	MG	DIF positive, IIF negative, anti-desmoglein 1 and 3, envoplakin, periplakin negative. GvHD-like changes in the skin biopsy sample	Tacrolimus, steroids, NB-UVB	Good response of oral mucosa erosions to steroids. Skin condition improvement over steroids + NB-UVB	Yatsuzuka et al. (29)
28	55	Female	B3, IVa	Generalized red keratotic papules tended to coalesce into plaques	PRCA	IIF negative GvHD-like changes in the skin biopsy sample	Steroid, cyclosporine A	Skin condition initially improved in steroids, recurred after steroids dose tapering. Cyclosporin A with no impact on skin lesions. Died of progressive multifocal leukoencephalopathy (positive JC virus in CSF)	Muramatsu et al. (36)
29	44	Female	IV	Erythematous papules on the skin of the trunk, mild diarrhea, serious liver dysfunction	MG	GvHD-like changes in the skin biopsy sample, unspecific features in the liver biopsy, could not rule out GvHD-like phenotype	Steroids, tacrolimus, IVIG	Died due to general deterioration	Mizutani et al. (15)
30	46	Female	B2 recurrent	Skin erythema, scaly eruptions of the trunk, face, lower extremities	MG	GvHD-like changes in the skin biopsy sample	Steroids	Improvement of the skin condition on high dose steroids	Pousa-Martinez et al. (26)
31	77	Female	B2	Pruritic scaly erythema on lower extremities that progressed to cover whole body	Hypogammaglobulinemia, Good syndrome	GvHD-like changes in the skin biopsy sample	Topical steroids, oral retinoid, NB-UVB	No improvement despite topical steroids with oral retinoid. NB-UVB with no impact. Skin condition improved after retinoid withdrawal. Patient alive 3 years after.	Fukushima et al. (23)
32	68	Female	B1, locally advanced	Erythematous, scaly papules of the face, buttocks, distal parts of upper extremities, oral mucosa erosions/lichenoid-like/candida-like changes	MG	GvHD-like changes of the skin and oral mucosa biopsy samples	Steroids	No improvement of skin and oral mucosa condition along with thymoma remission nor on steroids. Patient stable and alive 3 years after TAMA onset.	Presented case

MG, myasthenia gravis; GvHD, graft-versus-host disease; CHT, chemotherapy; RT-radiotherapy; NB-UVB, narrow band ultraviolet B; PUVA, psoralen + ultraviolet A; LFTs, liver function tests; PRCA, pure red blood cell aplasia.

In contrast to TAMA, where GvHD-like features are strictly required for diagnosis, PAMS demonstrated considerable symptom heterogeneity between patients, due to the variable manifestations of mucocutaneous lesions, e.g. pemphigus-like, bullous pemfigoid-like, erythema multiforme-like, GvHD-like and lichen planus-like forms (10). The uncertainty encountered during the presented diagnostic process resulted from the fact that GvHD-like dermatitis is common for both PAMS and TAMA. On the other hand, GvHD-like mucositis, typical for PAMS, was an undescribed but possible symptom in TAMA. Additionally, the mucocutaneous lesions did not improve in response to corticosteroid therapy; that stays in accordance with previous reports, where steroid therapy or palliative treatment of underlying neoplasm did not assure remission in TAMA, and only resulted in transient improvement in PAMS (37, 38). The present report is the first to discuss a patient with both an established TAMA diagnosis and GvHD-like mucositis. Hence, we suggest that GvHD-like mucositis should be placed in the spectrum of TAMA.

Scarce data exists on pathomechanisms of TAMA beyond the common background of cellular autoimmunity in thymoma e.g. errors in negative T-cells selection, decreased expression of autoimmune regulator – *AIRE* gene, overproduction of CD4+ and CD8+ single-positive and immature CD4+CD8+ double-positive T-cells, decreased Treg number and decreased major histocompatibility complex (MHC) class II expression (25, 32, 39, 40). Previous studies have found humoral reactions to predominate over cellular reactions in PAMS, and this is reflected in the diagnostic criteria. A few studies on PAMS have shown that tumors like thymoma, follicular dendritic cell sarcoma or B-cell lymphomas can secret antibodies against various proteins which could be a cornerstone in PAMS pathogenesis (41, 42). Hence, PAMS diagnosis requires an accumulation of antibodies in affected tissue; this can be confirmed by either DIF, positive rat bladder IIF, the presence of circulating antibodies directed mostly against plakin proteins family (desmoplakin 1, desmoplakin 2, envoplakin, periplakin) or against BP180, BP230, A2ML1. In contrary, expression of antibodies against desmoglein 1 and desmoglein 3 is characteristic rather of pemphigus vulgaris (PV), pemphigus foliaceus (PF) and paraneoplastic pemphigus (PNP) but not PAMS (10, 12, 43). Nevertheless, up to 16% of PAMS patients do not express circulating antibodies in ELISA assay (44). In some of these cases, the role of BP180 specific CD4+T-cells was postulated as possible cellular mechanism of autoimmunity, especially in GvHD-like and lichen planus-like PAMS manifestations (45–47). Taking that in mind, serological tests for exclusion of PAMS, PNP, PV and PF should be considered while establishing TAMA diagnosis.

A literature search on EMBASE, MEDLINE, PubMed and Cochrane databases with the terms *thymoma*, *remission* and *steroid* identified only 26 reports of thymoma patients demonstrating objective responses to corticosteroid monotherapy since 1952 (48–61). Out of these cases, five were reported to have complete remissions, while only one received a CT reassessment (57) and the remaining four were followed-up with X-ray examination only (48, 49, 56, 61). Spectacular responses were thought to be caused by an impact of corticosteroids on lymphocytic component of the tumor however, a few remissions in mixed and epithelial dominant tumors were also described (58). In most cases, corticosteroids were launched as palliation or for managing autoimmunity symptoms, which was also an indication in the presented patient (in majority for myasthenia gravis control – 11 cases, incidentally syndrome of inappropriate antidiuretic hormone secretion (SIADH) and PRCA were reported, no reports on TAMA nor PAMS). Nevertheless, an astonishing 47.1% objective response rate (ORR) was reported in a study of 17 patients with stage II-IVb thymomas to whom corticosteroids were administered prior to thymectomy. Aforementioned study showed 100% ORR in a WHO type B1 subgroup, that was also diagnosed in our patient, which may have been caused by apoptosis of immature double-positive CD4+CD8+ lymphocytes triggered by corticosteroids. However, the study also noted a reduction of the TUNEL-stained epithelial component of thymomas following steroid treatment, implicating a more complex relationship between corticosteroid therapy, tumor cellular composition and ORR (55).

The described patient demonstrates two extremely rare phenomena that can accompany thymomas: TAMA and spectacular tumor remission on corticosteroids. Moreover, TAMA presentation in this case was unprecedented due to its GvHD-like oral mucosa involvement that required additional differential diagnosis and exclusion of PAMS. Hence, we suggest that GvHD-like mucositis should be placed in the spectrum of TAMA.

## Data Availability Statement

The original contributions presented in the study are included in the article/**Supplementary Material**. Further inquiries can be directed to the corresponding author.

## Ethics Statement

Ethical review and approval was not required for the study on human participants in accordance with the local legislation and institutional requirements. In accordance with the Declaration of Helsinki, written consent for publication of this case description accompanied by test results and images was obtained from the patient.

## Author Contributions

EW, SD-S, RC, and PP designed and revised the manuscript. EW prepared case description and literature review and wrote the manuscript. MP conducted dermatologic evaluation and revised the manuscript. MB conducted histopathology analysis of skin and oral mucosa samples and revised the manuscript. All authors contributed to the article and approved the submitted version.

## Funding

The article was supported financially by Medical University of Lodz.

## Conflict of Interest

The authors declare that the research was conducted in the absence of any commercial or financial relationships that could be construed as a potential conflict of interest.

## Supplementary Material

Supplementary Table 1 Comparison of PAMS and TAMA diagnostic criteria, histopathology, laboratory tests results and treatment.

Supplementary Figure 1 A timeline of diagnosis and treatment of the patient.
