# Supplementary material for: Remission of Thymoma on Steroid Therapy in a Patient With Atypical Thymoma-Associated Multiorgan Autoimmunity: A Case Report and Literature Review
Source: Front Immunol. 2021 Apr 29;12:584703. doi: 10.3389/fimmu.2021.584703 (PMC8116704; doi:10.3389/fimmu.2021.584703)
Supplement: Supplementary Table 1 — Comparison of PAMS and TAMA diagnostic criteria, histopathology, laboratory tests results and treatment. [file Table_1.docx]

|  | PAMS | TAMA |
| --- | --- | --- |
| Diagnostic criteria | 1. Mucocutaneous manifestation, often with bronchial involvement 2. Identification of concurrent neoplasm (e.g. thymoma) 3. Laboratory evidence of humoral reaction against components of epithelial cell junctions 4. Progressive course, refractory to treatment, mostly fatal | 1. Thymoma confirmation 2. GvHD-like features in liver, colon and/or skin biopsy 3. No prior haematopoetic stem cell or organ transplantation |
| Histopathology | Suprabasilar acantholysis, subepidermal blister, dyskeratosis, keratinocyte necrosis, diffuse lymphocytic infiltration, interface vacuolar changes | Parakeratosis, dyskeratosis, basal layer destruction, intracellular oedema, focal appendages of skin destruction, subepidermal lymphocyte infiltration |
| Laboratory results | Positive for DIF, rat bladder IIF, antibodies against especially envoplakin/periplakin in ELISA testing | Subsequent to dermatitis, enterocolitis, hepatitis; does not require nor is excluded by DIF, IIF or PNP specific antibodies positivity |
| Treatment | IV immunoglobulins, corticosteroids, azathioprine, mycophenolate mofetil, cyclosporine, rituximab, phototherapy | |
|  | Dapsone, cyclophosphamide, methotrexate, thalidomide |  |
