# Supplementary figures and images for: Remission of Thymoma on Steroid Therapy in a Patient With Atypical Thymoma-Associated Multiorgan Autoimmunity: A Case Report and Literature Review
Source: Front Immunol. 2021 Apr 29;12:584703. doi: 10.3389/fimmu.2021.584703 (PMC8116704; doi:10.3389/fimmu.2021.584703)

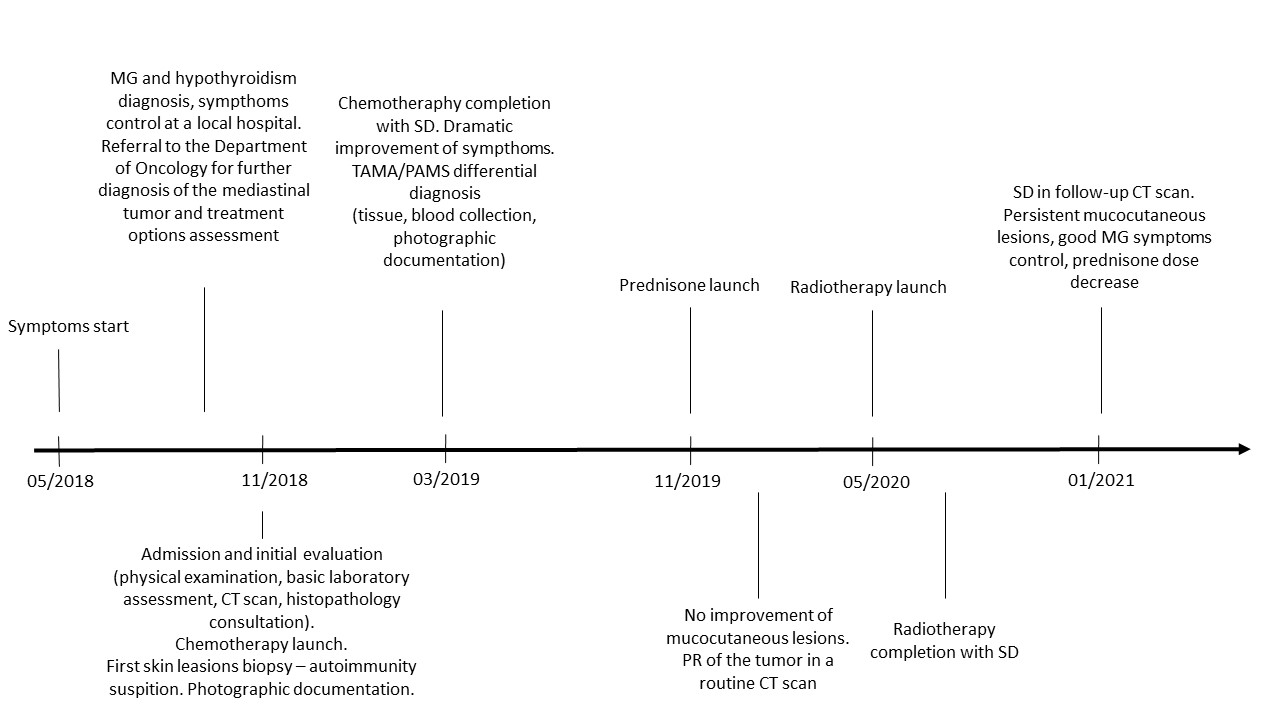

Supplement: Supplementary Figure 1 — A timeline of diagnosis and treatment of the patient. [file Image_1.jpeg]
